# The long noncoding RNA KCNQ1DN suppresses the survival of renal cell carcinoma cells through downregulating c-Myc

**DOI:** 10.7150/jca.29280

**Published:** 2019-08-19

**Authors:** Fan Yang, Qingjian Wu, Le Zhang, Wei Xie, Xiaoli Sun, Yan Zhang, Lei Wang, Qian Dai, Hua Yu, Qian Chen, Halei Sheng, Jing Qiu, Xiaomei He, Hongming Miao, Fengtian He, Kebin Zhang

**Affiliations:** 1 Central Laboratory, Xinqiao Hospital, Army Medical University (Third Military Medical University), Chongqing 400037, China;; 2 Department of Biochemistry and Molecular Biology, College of Basic Medical Sciences, Army Medical University (Third Military Medical University), Chongqing 400038, China;; 3 Department of Urology, Xinqiao Hospital, Army Medical University (Third Military Medical University), Chongqing 400037, China.; 4 Nursing division, Xinqiao Hospital, Army Medical University (Third Military Medical University), Chongqing 400037, China.

**Keywords:** KCNQ1DN, c-Myc, long non-coding RNA, renal cell carcinoma

## Abstract

**Background**: Long noncoding RNAs (lncRNAs) have been demonstrated to play essential roles in renal cell carcinoma (RCC). However, the role of lncRNA KCNQ1DN in RCC remains unclear.

**Methods**: The expression of KCNQ1DN in RCC and the corresponding adjacent tissues was measured by qPCR. RNA fluorescence in situ hybridization (FISH) assay, methylation analysis, reporter gene assays and functional tests were performed to reveal the effects of KCNQ1DN on RCC.

**Results**: In the present study, we found that lncRNA KCNQ1DN was notably decreased in RCC tissues and cell lines. RNA FISH assay showed that KCNQ1DN mainly localized to the cytoplasm. Methylation analysis revealed that the proximal region of KCNQ1DN promoter was hypermethylated in RCC tissues relative to the adjacent normal ones. Functional studies clarified that KCNQ1DN repressed the RCC cell growth and cell cycle progression. Mechanistically, KCNQ1DN inhibited the expression of c-Myc, which might further upregulate cyclin D1 and suppress p27 at mRNA and protein levels in RCC cells. Reporter gene assays revealed that the transcriptional activity of *c-Myc* promoter was inhibited by KCNQ1DN. The *in vivo* experiments in nude mice showed that KCNQ1DN overexpression dramatically repressed the growth of xenograft tumors and the expression of corresponding c-Myc.

**Conclusion**: These results indicated that KCNQ1DN inhibit the growth of RCC cells *in vitro* and *in vivo* through repressing the oncogene *c-myc,* suggesting that KCNQ1DN may serve as a novel target for the treatment of RCC.

## Introduction

Renal cell carcinoma (RCC), arising from renal tubular epithelial cells, is the most common malignancy of the kidney and accounts for more than 90% of adult renal malignancies^1^, ^2^. About 75%~80% of RCC is clear cell renal cell carcinoma (ccRCC), which is the most common subtype of RCC^1^. Despite advanced diagnosis, only about 20%~30% of RCC patients are diagnosed with metastasis. In addition, nearly 30% of patients undergoing nephrectomy will develop recurrence and metastasis^3^. Therefore, it is important to look for early diagnosis biomarkers and therapeutic targets.

Long noncoding RNAs (lncRNAs) are a class of transcripts longer than 200 nucleotides and have limited coding potential^4^. The functions of majority of lncRNAs areunknown but several studies have demonstrated that lncRNAs play critical roles in a variety of biological processes, including the epigenetic regulation, gene transcription regulation and post-transcriptional regulation^5^-^8^. LncRNAs are involved in many important biological processes in cancer such as cell apoptosis, cell cycle, cell survival, cell migration and so on^9^-^13^.

KCNQ1DN is a lncRNA, that has 1109 nucleotides and is located in chr11p15.5. In 2000, Xin et al showed that *KCNQ1DN* is an imprinting gene that is expressed in the maternal allele, but so far the functions of KCNQ1DN have not been identified^14^. In this study, we found that KCNQ1DN was downregulated in RCC tissues and cell lines, and this lncRNA inhibited cell growth and cell cycle progression of RCC cells via downregulating c-Myc. The *in vivo* studies in nude mice showed that overexpression of KCNQ1DN significantly repressed xenograft tumor growth and c-Myc expression. These findings demonstrated that the pathway 'KCNQ1DN/c-Myc' inhibits RCC cell growth, suggesting that this pathway may serve as a novel target for the treatment of RCC.

## Materials and Methods

### Patients and specimens

A total of 29 pairs of renal cancer specimens and corresponding non-tumor tissues were collected from patients who had undergone radical nephrectomy at the department of urology, Xinqiao Hospital, Army Medical University (Third Military Medical University). All the patient specimens were diagnosed of ccRCC by histopathological examination. The research was approved by the ethical committee of the Third Military Medical University of China, and each patient signed written informed consent before surgery. The clinic pathological characteristics of the patients are summarized in Table S1.

### Cell culture

RCC cell lines (ACHN, Caki-1, A498, 769-P, 786-O) and relative normal proximal tubule epithelial cell line HK-2 were purchased from the Cell Bank of Chinese Academy of Sciences (Shanghai, China). ACHN and A498 cells were cultured in MEM (Gibco, Carlsbad, CA, USA). 769-P and 786-O were cultured in RPMI 1640 (Gibco). Caki-1 and Caki-2 were cultured in McCoy's 5A (Gibco). The normal proximal tubule epithelial cell line HK-2 was cultured in DMEM/F-12 1:1 (Gibco). All the cells were cultured in 10% FBS (Gibco), at 37°C in a 5% CO2 humid incubator.

### RNA extraction and qPCR analysis

Total RNA was extracted from cells/frozen tissue specimens by Trizol reagent (Takara, Dalian, China). About 1 μg total RNA was reverse transcribed to cDNA using PrimeScript™ RT reagent Kit with gDNA Eraser (Takara) according to the manufacturer's protocol. qPCR was performed using the Power SYBR Green Master Mix kit (Thermo Fisher Scientific Inc, MA, USA). The results were standardized to the expression of β-actin. The primers were listed in Table S2.

### Transfection assay

RCC cells were transfected with siRNA oligonucleotides and plasmids using Lipofectamine 3000 (Invitrogen, USA) according to the manufacturer's instruction. Cells were seeded into culture plates and then transfected with siRNAs and plasmids by using Lipofectamine 3000. Forty-eight hours later, the cells were harvested for further studies. The siRNAs were purchased from Genepharma (Shanghai, China), and the corresponding sequences were listed in Table S3.

### RNA FISH

The RNA FISH probe mixture of KCNQ1DN, 18S or U6 RNA was synthesized and labeled with Cy3 from RiboBio. RNA FISH kit was purchased from RiboBio (Guangzhou, China). RNA FISH was performed as previously described^15^. The 6-diamidino- 2-phenylindole (DAPI, RiboBio) was used for nuclei counterstaining, and high resolution images were taken using a laser scanning confocal microscope (ZEISS, Jena, Germany).

### Quantitative methylation analysis

The website (http://www.ebi.ac.uk/Tools/seqstats/emboss_cpgplot/) was used to predict the CpG island. The primer-5′ (aggaagagagTTTGGGGTTTTAGAGTATTTGAGTG) and primer-3′ (cagtaatacgactcactatagggagaaggctACACAAAAACCCATTCTTCCTAACT) were designed to cover large region of the KCNQ1DN promoter CpG island. The selected amplicon was located in the promoter region (-1111/-556) of the gene. The mass spectra were collected using a MassARRAY Compact MALDI-TOF (Sequenom, BioMiao Biological Technology, Beijing, China) and the spectra's methylation ratios were generated by the EpiTYPER software (Sequenom, San Diego, CA).

### Cell proliferation assay

The cell proliferation was analyzed with CCK-8 (Dojindo Laboratories, Kumamoto, Japan) according to the manufacturer's protocol. Briefly, 2000 cells were seeded in triplicate in 96-well plates and given different treatments for the indicated times. Then the cell viability was assessed by measuring absorbance at 450 nm according to the manufacturer's guidelines.

### Plasmid generation

The DNA fragment encoding lncRNA KCNQ1DN was chemically synthesized and inserted into pcDNA3.1 (Invitrogen, Carlsbad, CA, USA) expression vector, and the resulting plasmid was named as pcDNA3.1-KCNQ1DN. The pEX-c-Myc vector and the empty vector pEX were purchased from Genepharma. qPCR assay was conducted to evaluate the expression of KCNQ1DN and c-Myc. The promoter region of the c-Myc gene F1(-2542/+4) and F2(-1384/+4) were cloned to the pGL3-Basic reporter vector (Promega, Madison, USA), and the recombinants were separately named pGL3-F1 and pGL3-F2.

### Flow cytometry assay

Differently treated cells were trypsinized and fixed with -20°C pre-cooling 70% ethanol at 4°C overnight. The cells were then washed three times with cold PBS. After incubation with RNase (0.1 mg/ml) at 37°C for 30 min, the cells were stained with propidium iodide (PI) 50mg/ml at room temperature for 15 minutes. PI-stained samples were analyzed with MoFlo XDP Beckman Coulter and the cell cycle distribution was calculated using ModFit LT software.

### Western blot assay

Cells were lysed to obtain proteins using RIPA. Western blots were performed to detect the specific protein expression levels as previously described [26]. Protein expression levels were normalized against GAPDH. Primary antibodies for c-Myc and GAPDH were purchased from Abcam (Cambridge, MA, USA). Anti-cyclin D1, p21 and p27 were purchased from Cell Signaling Technology (Boston, MA, USA).

### Dual-luciferase reporter assay

786-O cells were seeded in 96-well plates and grown to 70%~80% confluence. The cells were transiently transfected with siRNAs or expression plasmids. After 48h, the cells were lysed, and the firefly and renilla luciferase activities were determined using the Dual-Luciferase Reporter System (Promega, Madison, USA). The luciferase activity was normalized to pRL-TK activity. Data are represented as fold induction after normalizing the luciferase activity of the tested sample to the corresponding control sample.

### Establishment of stable-expressing cell lines

The DNA fragment encoding KCNQ1DN was synthesized by Sangon Biotech (Shanghai, China) and inserted into vector pGLV5 (Genepharma, Shanghai, China), and the resulting plasmid was named as pGLV-KCNQ1DN. The empty vector pGLV5 WAS named as pGLV-control. The packaged lentivirus separately containing the two plasmids above were used to infect 786-O cells at a multiplicity of infection (MOI) of 10, and then screened with puromycin. The resulting 786-O cell lines were named KCNQ1DN cell line (stable overexpression of KCNQ1DN) and control cell line.

### Xenograft experiments *in vivo*

All animal experiments were performed according to the protocol approved by the Animal Care and Ethics Committee of Third Military Medical University, Chongqing, China. Six-week old male BALB/c nude mice were randomly divided into two groups, with 6 mice in each group. 786-O cells stably transfected with pGLV-KCNQ1DN or pGLV-control were injected subcutaneously on the right flanks of mice (5×10^6^ cells per mouse) for 30 days. Then the mice were sacrificed by cervical dislocation, and the xenograft tumors were removed and weighed. The target molecules in the xenograft tumor tissues were detected by Western blot and qPCR.

### Statistical analysis

All data were expressed as Mean ± standard deviation. All statistical analyses were carried out with Statistical Package for the Social Sciences (SPSS), version 17.0 (SPSS Inc., Chicago, IL, USA). P < 0.05 was considered statistically significant.

## Results

### KCNQ1DN is downregulated in RCC tissues and cell lines

**Figure 1:**
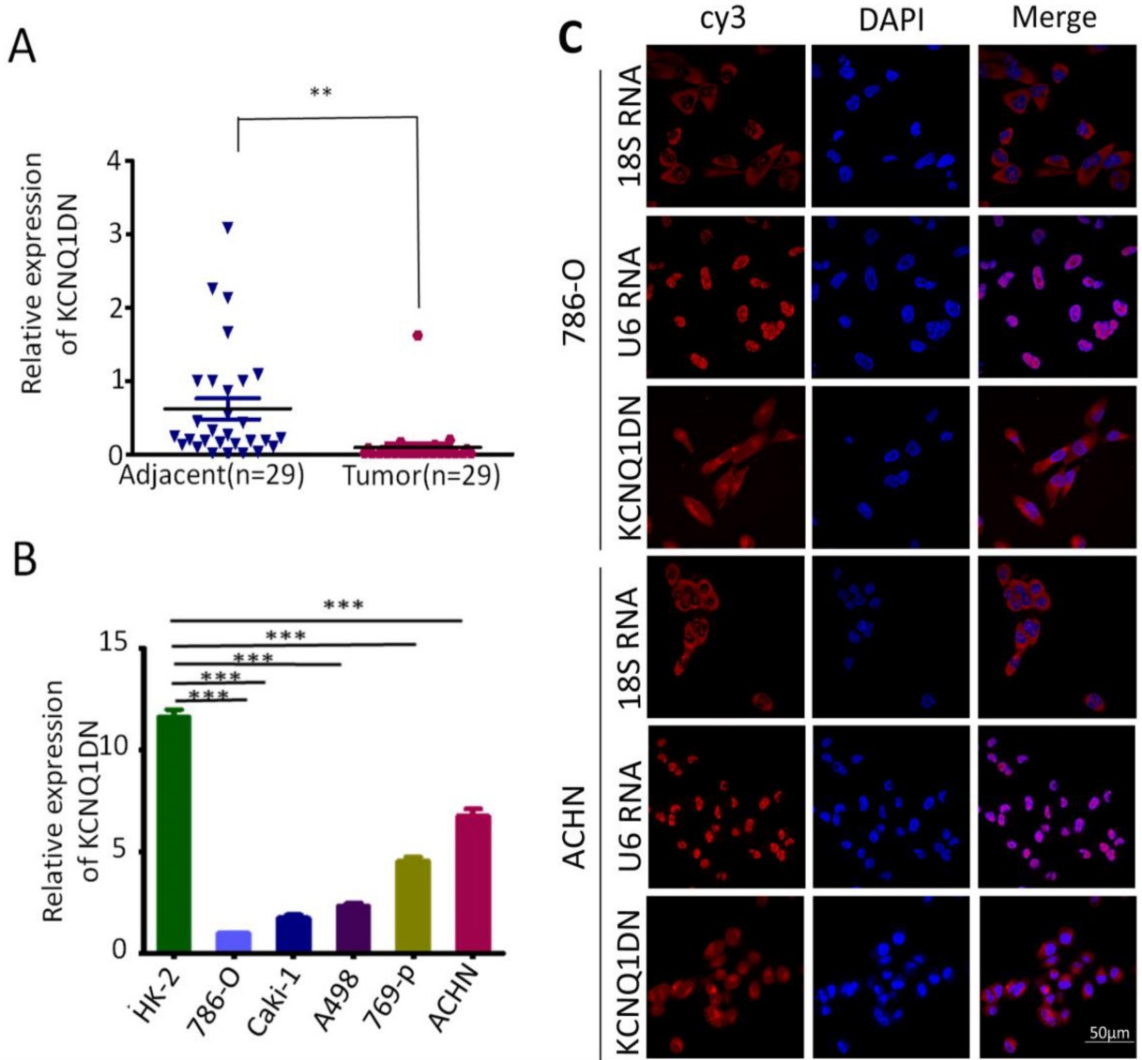
KCNQ1DN is downregulated in RCC tissues and cell lines. ***A,*** qPCR analysis of KCNQ1DN expression in 29 pair-wise ccRCC tissues and the corresponding adjacent non-tumor tissues, taking β-actin as a control. Tumor: ccRCC tissues, Adjacent: adjacent non-tumor tissues.*** B,*** qPCR analysis of KCNQ1DN expression in RCC cell lines (786-O, Caki-1, A498, 769-P and ACHN) and the relative normal proximal tubule epithelial cell line HK-2. ***C,*** FISH assay was performed to detect the distribution of KCNQ1DN in RCC cells. The 18S RNA and U6 RNA were separately used as cytoplasmic and nuclear RNA controls. The KCNQ1DN probe mix and control RNA probe mix were labeled with Cy3. DAPI was used to counterstain the nuclei. The high resolution images were captured with a laser scanning confocal microscope. **P <0.01, ***P <0.001.

Previous studies have shown that the expression of KCNQ1DN is decreased in Wilms' tumors^14^. To investigate the role of KCNQ1DN in RCC, the expression of KCNQ1DN in 29 pair-wise ccRCC tissues and the corresponding adjacent non-tumor tissues were examined. As shown in Figure 1A, KCNQ1DN was significantly decreased in RCC tissues. Moreover, the level of KCNQ1DN in RCC cell lines (ACHN, Caki-1, A498, 769-P and 786-O) was significantly lower compared to HK-2 cells (Figure 1B). RNA FISH assay revealed that KCNQ1DN was mainly distributed in the cytoplasm, and a small portion of KCNQ1DN distributed in the nucleus (Figure 1C). Taken together, these findings suggested that KCNQ1DN may play an essential role in RCC development and progression, and this lncRNA may serve as a novel potential biomarker and therapeutic target for RCC.

### KCNQ1DN gene promoter region is hypermethylated in RCC tissues

**Figure 2:**
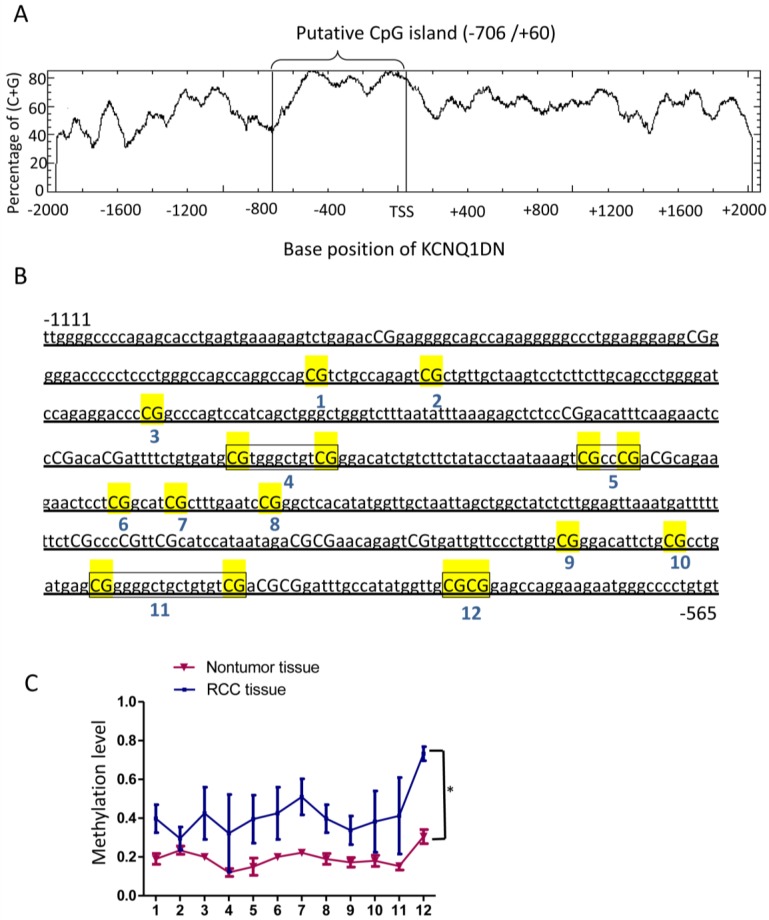
KCNQ1DN gene promoter region is hypermethylated in RCC tissues. ***A,***A CpG island spanning from -706 to +60 of *KCNQ1DN* gene was identified by EMBOSS CpGplot program (http://www.ebi.ac.uk/Tools/seqstats/emboss_cpgplot/). ***B****and** C***, The translation start site (TSS) is position +1 and the rest of the sequence is numbered relative to it. Twelve CpG sites were detected in all of the samples and then compiled. N=4; *P <0.05.

To detect the mechanism of KCNQ1DN downregulation in RCC tissues, DNA methylation of the promoter region of *KCNQ1DN* gene was analyzed. The website (http://www.ebi.ac.uk/Tools/seqstats/emboss_cpgplot/) was conducted to predict the CpG island of KCNQ1DN promoter (-2000/+2073). One CpG island with 767 bp (-706/+60) was found (Figure 2A). Quantitative methylation analysis was further performed on *KCNQ1DN* gene promoter (-1111/-565) with an amplicon covering 22 CpG sites in non-tumor and RCC tissues. Twelve CpG sites were detected in all of the samples and statistics to these data were compiled (Figure 2B). These data showed that RCC tissues have higher methylation on the 12 CpG sites (Figure 2C).

### KCNQ1DN inhibits the growth and cell cycle progression of RCC cells

**Figure 3:**
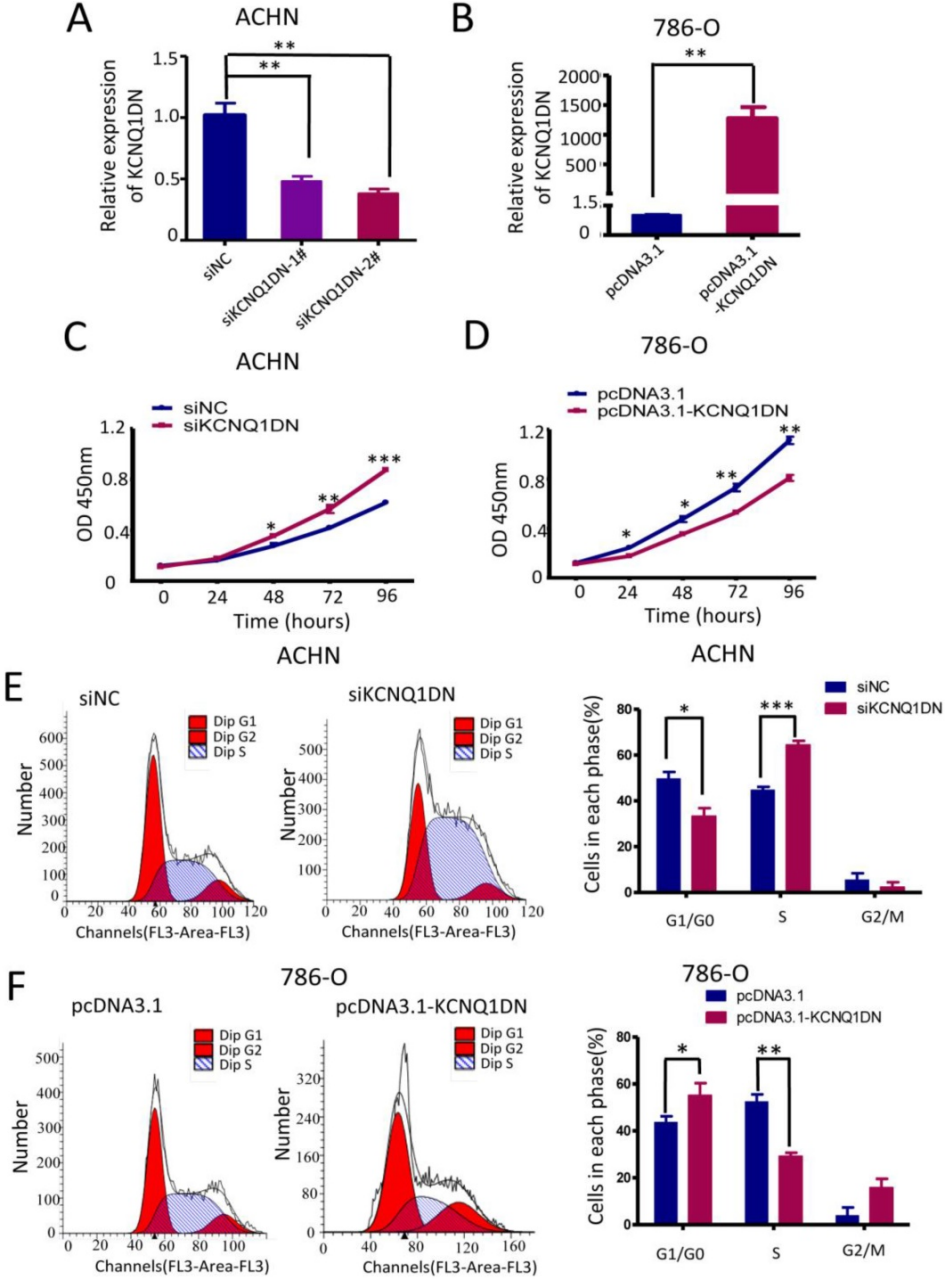
KCNQ1DN inhibits the growth and cell cycle progression of RCC cells. 786-O and ACHN cells were transfected with the indicated siRNAs or plasmids for 48 h, followed by qPCR ***(A, B)***, CCK-8 (***C, D)*** and flow cytometry (***E, F)*** analyses. siNC: control siRNA; siKCNQ1DN: siRNA for KCNQ1DN; pcDNA3.1-KCNQ1DN: expression plasmid of KCNQ1DN; *P<0.05, **P<0.01, ***P<0.001.

To study the function of KCNQ1DN, both loss- of-function and gain-of-function approaches were used in ACHN and 786-O cells (Figure 3A, B). The results showed that knockdown of KCNQ1DN by siRNAs significantly promoted cell survival (Figures 3C), while overexpression of KCNQ1DN dramatically suppressed RCC cell growth (Figures 3D). Cell cycle analysis revealed that knockdown of KCNQ1DN enhanced the G1 progression in ACHN cells (Figure 3E), while overexpression of KCNQ1DN resulted in cell cycle arrest in G1 phase in 786-O cells (Figure 3F). These findings indicated that KCNQ1DN can repress the growth and cell cycle progression of RCC cells *in vitro*.

### KCNQ1DN regulates the levels of cyclin D1 and p27 in RCC cells

**Figure 4:**
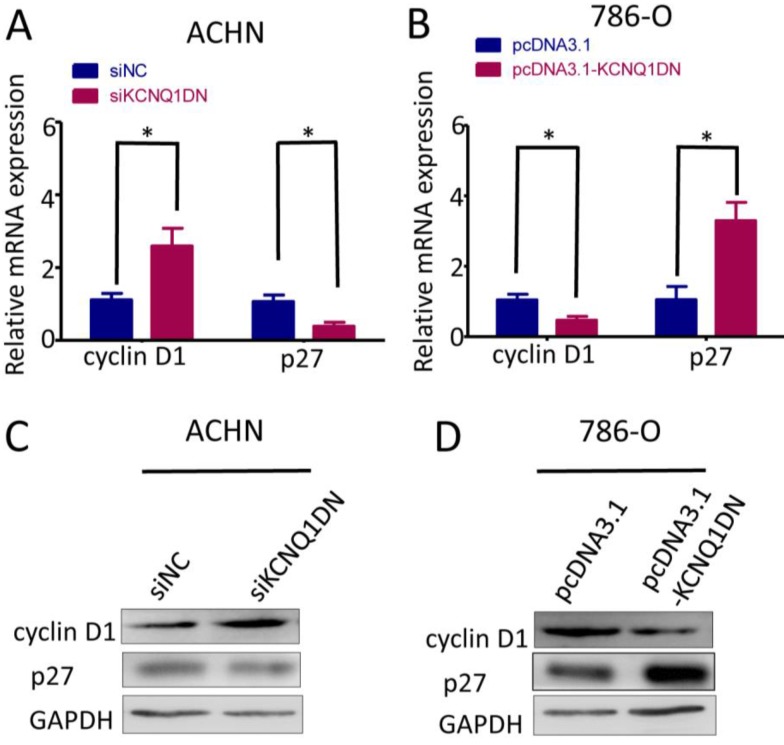
KCNQ1DN regulates the levels of cyclin D1 and p27 in RCC cells. 786-O and ACHN cells were transfected with the indicated siRNAs or plasmids for 48 h, then the mRNA and protein levels of cyclin D1 and p27 were detected by qPCR ***(A, B)*** and Western blot ***(C, D)***. siNC: control siRNA; siKCNQ1DN: siRNA for KCNQ1DN; pcDNA3.1-KCNQ1DN: expression plasmid of KCNQ1DN; *P<0.05.

To explore how KCNQ1DN suppresses cell cycle progression of RCC cells, we detected cyclin D1 and p27, which regulates the cell-cycle transition during G1/S. As shown in Figure 4A and 4C, knockdown of KCNQ1DN in ACHN cells markedly upregulated cyclin D1 but downregulated p27 at mRNA and protein levels. Figure 4B and 4D showed that overexpression of KCNQ1DN in 786-O cells dramatically decreased cyclin D1 but increased p27 at the mRNA and protein levels. These results demonstrated that KCNQ1DN regulates the expression of cyclin D1 and p27 in RCC cells.

### KCNQ1DN downregulates c-Myc by inhibiting the transcriptional activity of its gene promoter

**Figure 5:**
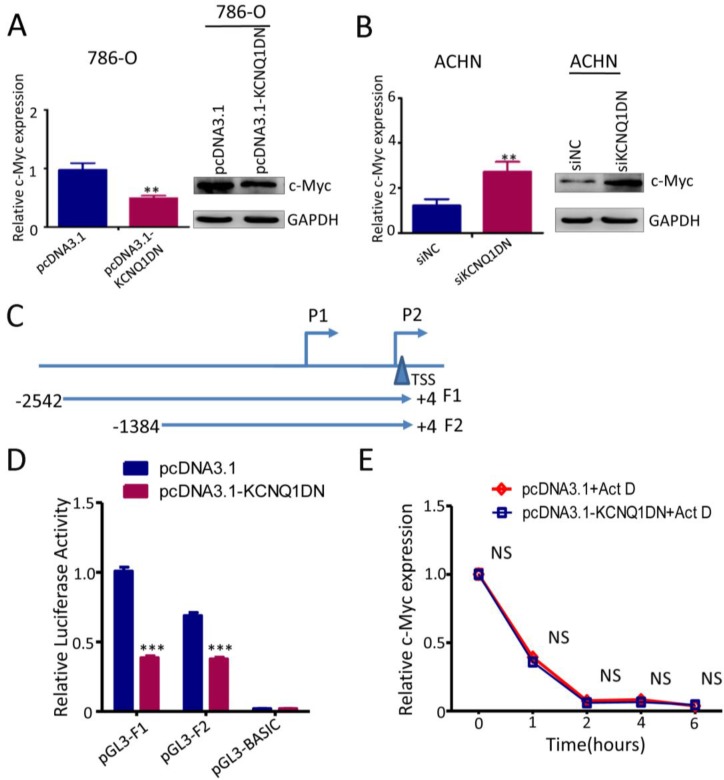
KCNQ1DN downregulates c-Myc by inhibiting the transcriptional activity of its gene promoter. ***A, B,*** 786-O and ACHN cells were transfected with the indicated siRNAs or plasmids for 48h, then the mRNA and protein levels of c-Myc were measured by qPCR and Western blot. ***C,***sketch map of the recombinant plasmid construction. ***D,***786-O cells were co-transfected with pGL3-F1 (or pGL3-F2 or control vector pGL3-basic), pRL-TK and the plasmid pcDNA3.1-KCNQ1DN (or control plasmid pcDNA3.1) for 48h. Then the firefly and renilla luciferase activities were measured using the dual-luciferase reporter system. The ratio of firefly/renilla luminescence for each well was calculated, and the test sample ratio was normalized against the control ratio. ***E,***After transfected with the indicated plasmids for 48 h, the 786-O cells were treated with actinomycin D (Act D, 10 μg/ml) for the indicated times. Then the expression level of c-Myc was detected by qPCR. siNC: control siRNA; siKCNQ1DN: siRNA for KCNQ1DN; pcDNA3.1-KCNQ1DN: expression plasmid of KCNQ1DN; **P<0.01, ***P<0.001.

It has been reported that c-Myc plays an essential role in promoting cell proliferation and cell cycle progression, and its two direct target genes* cyclin D1* and* p27* are involved in regulating the cell cycle progression. To explore whether KCNQ1DN could inhibit c-Myc expression, the mRNA and protein levels of c-myc were detected under the condition of overexpression or knockdown of KCNQ1DN. As shown in Figures 5A and 5B, overexpression of KCNQ1DN in RCC cells significantly reduced c-Myc expression at transcriptional and translational levels (Figures 5A), while knockdown of KCNQ1DN remarkably elevated c-Myc expression at mRNA and protein levels (Figure 5B). Moreover, Figure S1 showed that silencing of c-Myc dramatically attenuated KCNQ1DN-knockdown induced survival enhancement of RCC cells, while ectopic expression of c-Myc markedly alleviated KCNQ1DN- overexpression induced growth inhibition of RCC cells. Additionally, the DNA fragments of *c-Myc* gene promoter region F1(-2542/+4) and F2(-1384/+4) were cloned into vector pGL3-BASIC (Figure 5C), and the dual-luciferase reporter assays were performed. As shown in Figure 5D, overexpression of KCNQ1DN significantly decreased the transcriptional activity of *c-Myc* gene promoter. The actinomycin D (Act D) assay showed that the degradation of c-Myc mRNA was not influenced by overexpression of KCNQ1DN (Figure 5E), indicating that KCNQ1DN does not downregulate c-Myc by reducing its mRNA stability. Collectively, these data demonstrated that KCNQ1DN downregulates the expression of c-Myc via inhibiting the transcriptional activity of *c-Myc* gene promoter.

### Overexpression of KCNQ1DN represses xenograft RCC growth and c-Myc expression *in vivo*

**Figure 6:**
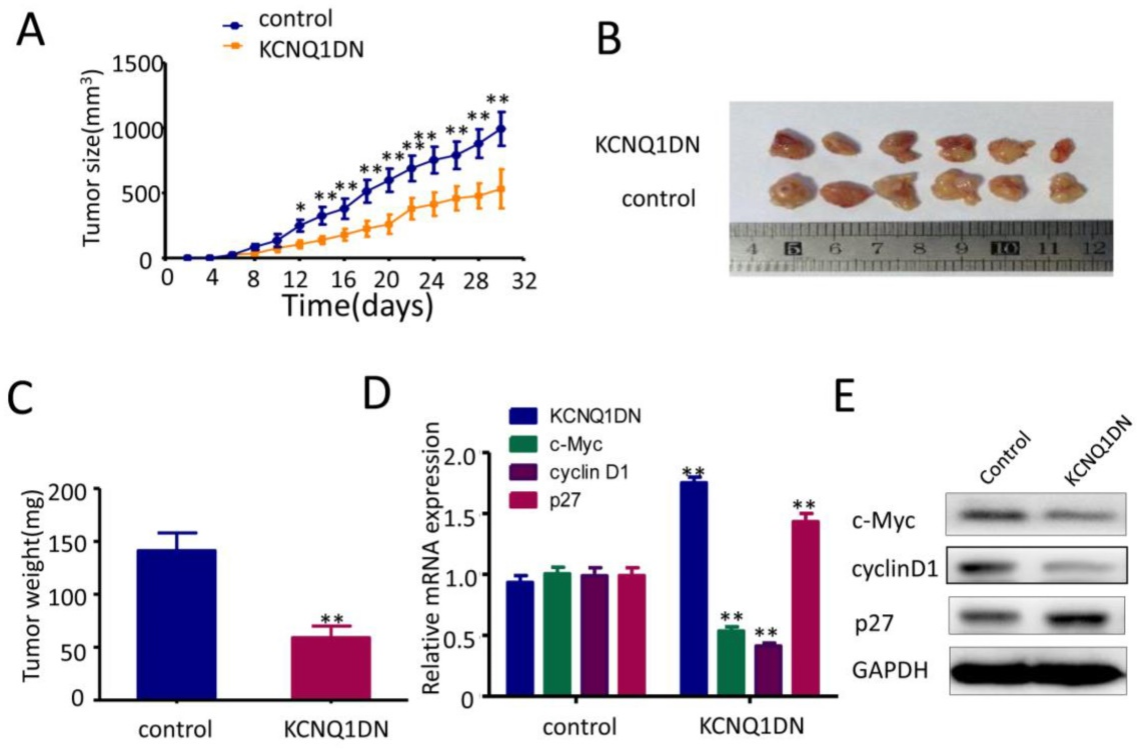
Overexpression of KCNQ1DN inhibits xenograft RCC growth and c-Myc expression *in vivo*. Six-week-old male nude mice were randomly divided into two groups (n=6 per group). Then 5×10^6^ KCNQ1DN cells (786-O cells with KCNQ1DN stable expression) and the corresponding control cells were separately injected subcutaneously into the right flanks of mice. ***A,***Xenograft tumor size was monitored every 2 days (tumor volume = width^2^×length×1/2) in 30 days. ***B, C,*** The mice were sacrificed, and the RCC xenografts were excised for the comparisons of tumor sizes ***(B)***and weights ***(C)***. ***D, E,*** The indicated molecules were examined by qPCR ***(D)*** and Western blot ***(E)***. *P< 0.05, **P< 0.01.

As shown in Figures 6A and 6B, overexpression of KCNQ1DN markedly reduced the sizes and weights of RCC xenograft in nude mice. qPCR (Figure 6D) and Western blot (Figure 6E) assays showed that overexpression of KCNQ1DN notably downregulated c-Myc and it downstream target cyclin D1 in RCC xenografts, but dramatically upregulated the c-Myc downstream target p27. Taken together, the tumor-bearing studies in nude mice indicated that overexpression of KCNQ1DN inhibits the growth of xenograft RCC and the expression of c-Myc *in vivo*.

## Discussion and Conclusion

RCC is the most common malignant neoplasm of the kidney. The global incidence of RCC has been increased at the rate of 2% per year over the past two decades^2^.For early stage RCC, partial nephrectomy is generally used as the standard approach to remove localized RCC with a good prognosis. However, The 5-year survival rate of metastatic RCC is only 10%^16^. Therefore, it is necessary to identify novel prognostic biomarkers and therapeutic targets to improve the detection and the treatment of RCC.

Global hypomethylation and regional hypermethylation can lead to aberrant expression of genes and activation of protooncogenes^17^, ^18^. In this study, KCNQ1DN was found to be downregulated in RCC tissues and cell lines. Many tumor suppressor genes have been reported to be partially or completely silenced from hypermethylation of their promoters sites^19^.The role of DNA methylation in RCC has remained an area of research interest over the past decade, and various genes are epigenetically altered in RCC by DNA methylation^19^-^21^. In this study, methylation analysis suggested that *KCNQ1DN* promoter hypermethylation could be one of the factors for low expression of KCNQ1DN in RCC. The p15.5 region of chromosome 11 in human genome contains the highest density of imprinted genes, which is divided into H19-TH and ASCL2-OSBPL5 gene cluster. Each block is independently regulated by imprinting control regions (ICRs) through differentially methylated regions (DMRs)^22^. However, the details of the mechanism need for further studies.

c-Myc is a conventional transcriptional factor. By binding to the *cis-*regulatory element E-box (CACGTG) of its transactivated genes, c-Myc plays an essential role in the regulation of many biological processes including cell cycle, cell proliferation, apoptosis and cell metabolism^23^-^25^. c-Myc has been reported to be upregulated in a variety of carcinomas including RCC. Both cyclin D1 and the CDK inhibitor p27 are important targets of c-Myc in cell cycle^26^, ^27^. In this study, we revealed that KCNQ1DN inhibited the growth and cell cycle progression of RCC cells via regulating c-Myc and its downstream targets cyclin D1 and p27, indicating that the pathway 'KCNQ1DN /c-Myc' is the possible mechanism by which KCNQ1DN represses RCC cell growth and cell cycle progression. However, the detailed mechanisms involved in the KCNQ1DN induced downregulation of* c-Myc* promoter activity are still not clear and more studies are required. In conclusion, the results of this work demonstrated for the first time that KCNQ1DN is decreased in RCC tissues and cell lines, and this lncRNA inhibits the growth and cell cycle progression of RCC cells by suppressing c-Myc. The *in vivo* studies in nude mice showed that overexpression of KCNQ1DN represses the growth of xenograft RCC and the expression of c-Myc. These findings revealed that the'KCNQ1DN/c-Myc' pathway inhibits RCC cell growth, suggesting that this pathway may serve as a novel target for RCC treatment.

## Supplementary Material

Supplementary figures and tables.
