# Supplementary material for: The long noncoding RNA KCNQ1DN suppresses the survival of renal cell carcinoma cells through downregulating c-Myc
Source: J Cancer. 2019 Aug 19;10(19):4662–70. doi: 10.7150/jca.29280 (PMC6746116; doi:10.7150/jca.29280)
Supplement: Supplementary file 1 — Supplementary figures and tables. [file jcav10p4662s1.pdf]

Supplementary tables

Table S1 The clinic pathological factors of ccRCC patients

Characteristics	Expression of KCNQ1DN		*P*-value[1]
	Low（15）	High（14）	
Sex			0.81
Male	9	9	
Female	6	5	
Age			0.60
≤60	10	8	
＞60	5	6	
Histological grade			0.92
Low or undiffer	12	11	
Middle or high	3	3	
TNM stage			0.91
I/II	11	10	
III/IV	4	4	

[1]Chi-square test.

Table S2. The primer sets used in qPCR

Targets	Forward primers (5′-3′)	Reverse primers (5′-3′)
h c-Myc	GCAGCTGCTTAGACGCTGGA	CGCAGTAGAAATACGGCTGCAC
h p27	AAATGTTTCAGACGGTTCCC	CATTCCATGAAGTCAGCGATA
h Cyclin D1	CGCCCCACCCCTCCAG	CCGCCCAGACCCTCAGACT
h KCNQ1DN	GCTGGGGTCTTCTCCTGA	CTCTTGTTTTGCTTTCTTTCC
h β-actin	GTGAAGGTGACAGCAGTCGGTT	GAAGTGGGGTGGCTTTTAGGA

h: human

Table S3. The sequences of siRNAs

siRNAs	Forward 5′—3′
siKCNQ1DN-1#	GCUGCCUUUGGACCAGAUUTT
siKCNQ1DN-2#	GCCCUUCUGAGAAAGCAAUTT
siNC	UUCUCCGAACGUGUCACGUTT

Figure S1

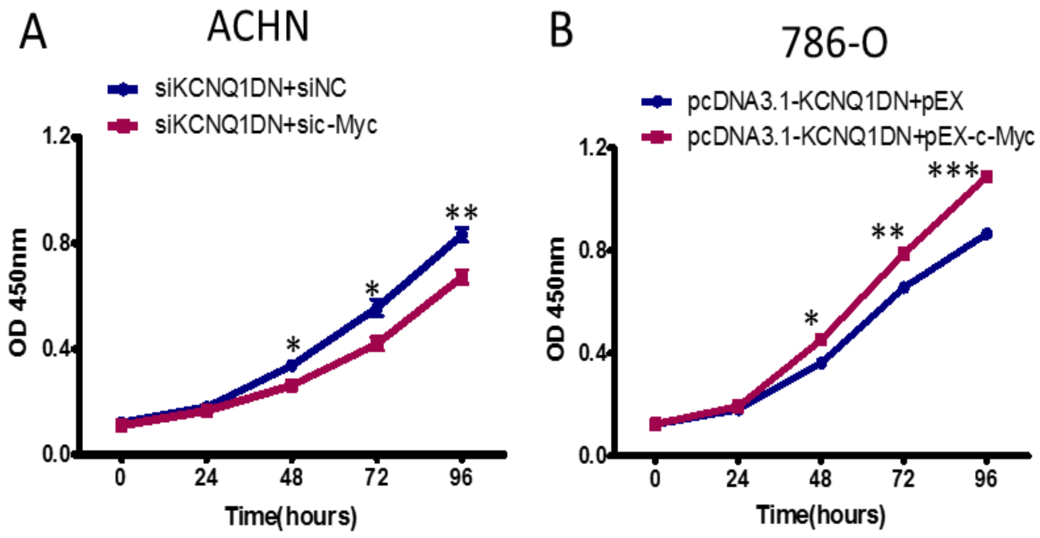

Figure S1, KCNQ1DN inhibits the survival of RCC cells through downregulating c-Myc. ACHN ***(A)*** and 786-O ***(B)*** cells were transfected with the indicated siRNAs or plasmids for 48h. The cell viability was determined by CCK-8 assay***s***. siNC: control siRNA; siKCNQ1DN: siRNA for KCNQ1DN; sic-Myc: siRNA for c-Myc; pcDNA3.1-KCNQ1DN: expression plasmid of KCNQ1DN; pEX-c-Myc: expression plasmid of c-Myc; *P<0.05, **P<0.01, ***P<0.001
